# Decline of Tuberculosis Burden in Vietnam Measured by Consecutive National Surveys, 2007–2017

**DOI:** 10.3201/eid2703.204253

**Published:** 2021-03

**Authors:** Hai Viet Nguyen, Hoa Binh Nguyen, Nhung Viet Nguyen, Frank Cobelens, Alyssa Finlay, Cu Huy Dao, Veriko Mirtskhulava, Philippe Glaziou, Huyen T.T. Pham, Petra de Haas, Edine Tiemersma

**Affiliations:** National Tuberculosis Programme, Hanoi, Vietnam (H.V. Nguyen, H.B. Nguyen, N.V. Nguyen, C.H. Dao, H.T.T. Pham);; Amsterdam University Medical Centers, Amsterdam, the Netherlands (H.V. Nguyen, F. Cobelens);; US Centers for Disease Control and Prevention, Vietnam Office, Hanoi (A. Finlay);; KNCV Tuberculosis Foundation, Den Haag, the Netherlands (V. Mirtskhulava, P. de Hass, E. Tiemersma);; World Health Organization, Geneva, Switzerland (P. Glaziou).

**Keywords:** Bacteria, frequentist inference, Mycobacterium tuberculosis, national prevalence surveys, respiratory infections, sputum smear microscopy, tuberculosis and other mycobacteria, Vietnam, Vietnam National TB Program

## Abstract

Vietnam, a high tuberculosis (TB) burden country, conducted national TB prevalence surveys in 2007 and 2017. In both surveys participants were screened by using a questionnaire and chest radiograph; sputum samples were then collected to test for *Mycobacterium tuberculosis* by smear microscopy and Löwenstein-Jensen culture. Culture-positive, smear-positive, and smear-negative TB cases were defined by laboratory results, and the prevalence of tuberculosis was compared between the 2 surveys. The results showed prevalence of culture-positive TB decreased by 37% (95% CI 11.5%–55.4%), from 199 (95% CI 160–248) cases/100,000 adults in 2007 to 125 (95% CI 98–159) cases/100,000 adults in 2017. Prevalence of smear-positive TB dropped by 53% (95% CI 27.0%–69.7%), from 99 (95% CI 78–125) cases/100,000 adults to 46 (95% CI 32–68) cases/100,000 adults; smear-negative TB showed no substantial decrease. Replacing microscopy with molecular methods for primary diagnostics might enhance diagnosis of pulmonary TB cases and further lower TB burden.

Despite global progress in preventing and controlling tuberculosis (TB), reducing worldwide burden has fallen short of the World Health Organization’s (WHO) End-TB elimination target (*1*). However, in most high-burden countries, estimates of burden and its trend over time are derived indirectly (*1*). One main factor that impedes global efforts to estimate the effects of and eliminate TB is the gap in case detection. In most high-burden lower-middle income countries, an unknown proportion of incident TB remains undiagnosed and unreported (*2*). To estimate TB incidence, WHO has applied the results of nationwide prevalence surveys to a model of disease duration distribution, among other estimation methods (*3*). These surveys were administered to determine a country’s gaps in detecting TB cases, help plan interventions, and estimate the resources required (*4*). Prevalence surveys measure TB burden directly; surveys that are repeated have particular strategic importance. Comparing TB prevalence over time enables public health authorities to assess the trend in burden and therefore to evaluate the effect of TB control interventions between surveys and develop policies to guide future actions (*5*).

Vietnam is among the 30 countries with the highest burden of TB in the world (*6*). In 2006–2007, the Vietnam National TB Program (NTP) conducted the first national TB prevalence survey, which identified 307 (95% CI 249–366) pulmonary TB cases/100,000 adult participants (*7*). Since then, to reduce the TB burden in Vietnam NTP has applied a broad package of TB control interventions (*8*). In addition to strengthening routine TB care and treatment, NTP has introduced new TB diagnostics, TB drugs, and treatment regimens for multidrug-resistant TB patients, preventive treatment for children ≤5 years of age living with ≥1 additional TB patients, household contact tracing, and active case finding (*9*,*10*). Although after 2007 the TB notification rate in Vietnam declined (*11*), it remained unknown to what extent this represented a decrease in TB burden.

In 2017–2018, NTP administered a second national TB prevalence survey in Vietnam, which showed 322 (260–399) TB cases/100,000 adults (*12*). These findings suggested no change in burden over the 10-year period. However, to be in line with WHO recommendations, the second survey used TB screening procedures and diagnostics that were state-of-the-art in 2017–2018 but not available in 2006–2007 (*13*). These methods differed from those used in the first survey, potentially affecting estimates of trends in prevalence. To enable direct comparisons, we also analyzed data from the second survey using screening and diagnostic methods similar to those used in the first survey. To directly measure the change in TB burden over time and the effect of control interventions in Vietnam, as much as possible we compared the results of the 2 surveys based on the same methods with regard to sampling, screening, laboratory testing, case definitions, and statistical adjustments. Our primary comparison considered the adjusted prevalence of culture-positive TB; secondary comparisons included adjusted and unadjusted prevalence of smear-positive TB and smear-negative TB.

This study was given scientific and ethics approval by the Institutional Review Board of the Vietnam National Lung Hospital, under approval letter number 62/17/CTHĐKH-ĐĐ. The risks and benefits of the study were explained to participants and each signed a written informed consent. All of those with >1 positive test result for TB were referred for TB treatment; we followed up to verify that they received adequate health care and treatment.

## Methods

### Study Design and Population

Both the first and second surveys were cross-sectional studies using multistage cluster sampling. The sample size of both surveys was based on the estimated prevalence of TB in the country at the time of study design (*3*). Also, a frequentist approach was applied in the second survey (*14*) to ensure a large enough sample for finding a difference in TB prevalence between the 2 surveys of >25%, with a power of 80%.

Sampling methods were similar between the 2 surveys, except for small details in the sampling frames, enumeration, and inclusion criteria (Appendix Table 1). The sampling frame used in the second survey did not include stratification, whereas the sampling frame in the first survey was stratified by urban, rural, and remote areas. Enumeration criteria differed slightly; the first survey included all adult residents (≥15 years old) who had lived in households of the selected clusters for >3 months, but the duration used in the second survey was ≥2 weeks. All enumerated persons who met the inclusion criteria were considered eligible for the second survey, but for the first survey, they also had to indicate that they planned to attend the survey site for screening.

### Screening and Diagnostic Procedures

For both surveys, all participants were screened for TB based on self-reported symptoms and chest radiograph results. Those who reported TB symptoms, had TB treatment history <2 years before the respective survey, or had chest radiograph abnormalities consistent with TB were considered screen-positive and were eligible for sputum collection and examination. For the second survey, all chest radiographs were digital, whereas in the first survey, only two thirds of the clusters used digital chest radiographs (Appendix Table 2). The criteria for defining screen-positive participants based on symptoms differed slightly between the 2 surveys. In the first survey only those reporting productive cough for >2 weeks were considered positive on their symptom screens. In the second survey, all participants reporting any cough lasting >2 weeks, as well as pregnant women reporting any cough of any duration, were considered to have a positive symptom screen. To compare the 2 surveys, we only considered participants screen-positive if they reported productive cough for >2 weeks or had a history of TB treatment <2 years before the survey (Appendix Table 3).

Recommended laboratory methods for prevalence survey estimates were followed in each survey, but guidelines have changed over time (*14*). The first survey used sputum smear microscopy and Löwenstein-Jensen (LJ) solid culture assays; the second survey used the molecular assay Xpert MTB/RIF (Cepheid, https://www.cepheid.com) and BD MGIT BACTEC 960 liquid culture (https://www.bd.com). To ensure comparable laboratory results between the surveys, for the second survey we also conducted sputum smear microscopy and LJ solid culture on sputum samples (Appendix Table 2). Sputum sample processing was slightly different between surveys. For the first survey we used 4% NALC-NaOH to decontaminate the specimen before LJ culture; for the second survey we used the modified Petrov method with 3% NALC-NaOH for decontamination.

### Case Definition

TB case definitions used for comparison purposes were based solely on laboratory results in which each screen-positive participant had 1 early morning sputum sample tested for TB by sputum smear microscopy and LJ culture in designated survey laboratories. Screen-positive participants whose result from sputum smear was positive for acid-fast bacilli and whose LJ culture grew *Mycobacterium tuberculosis* were defined as smear-positive TB cases, whereas those who had a negative sputum smear result and LJ culture grew *Mycobacterium tuberculosis* were defined as smear-negative. Culture-positive TB cases had either smear-negative or smear-positive TB.

### Data Analysis

We combined data from the 2 surveys into 1 database, assigning a unique personal identification code for each observation, and adapted the variables needed to compare the 2 surveys. Similar to what we reported for the second survey, data analysis was conducted on the combined database, with updated inclusion criteria and case definitions, as stated (*12*). The adjusted analysis involved multiple imputation by chained equation for missing data, including sputum smear and LJ culture results, and inverse probability weighting as recommended by WHO (*15*). To ensure the comparability of the 2 surveys and adjust for the relative sampling probability of each participant in both surveys, we applied poststratification using population data for Vietnam estimated by the General Statistics Office of Vietnam in 2007 and 2017 (*16*).

We assessed statistical differences in characteristics between the 2 surveys by χ^2^ test and logistic regression. We calculated point estimates and 95% CIs for TB prevalence in Stata version 14.0 (StataCorp, https://www.stata.com) using the *mim* and *svy* commands with *pweights* specified to adjust for design effect. We derived 95% CIs and p values using Rubin’s rules (*17*). To assess whether TB prevalence had changed between the 2 surveys, we calculated a prevalence difference on the combined dataset using Stata’s *epitab* module (StataCorp), including the outcome “TB case Yes/No” as the dependent variable and first or second survey as the explanatory variable.

## Results

**Figure 1:**
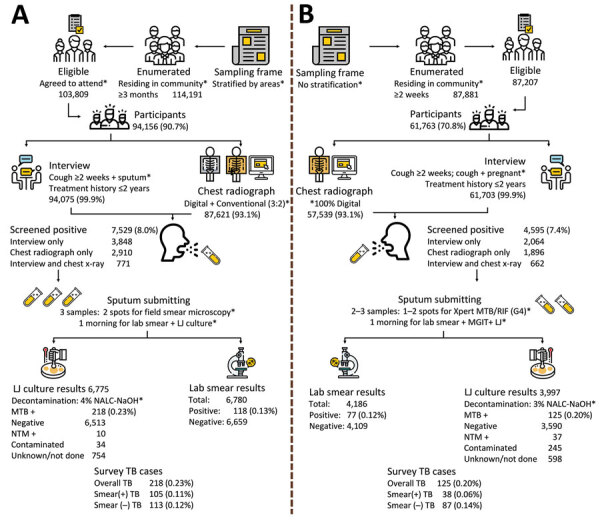
Summary results and comparison between the first (A) and second (B) national TB prevalence surveys in Vietnam, 2007 and 2017. Asterisk (*) indicates differences in methods between the 2 surveys. MTB, *Mycobacterium tuberculosis*; NTM, nontuberculosis mycobacteria; TB, tuberculosis; +, positive; –, negative.

There were 94,156 participants (90.7% of the eligible survey population) in the first survey and 61,763 (70.8% of the eligible survey population) in the second. Among participants in the first survey, 99.9% were screened for TB symptoms and had chest radiographs; in the second survey, 93.1% were screened and had radiographs. Among all survey participants, in the first survey 7,529/94,156 (8.0%) tested screen-positive; in the second, 4,595/61,763 (7.3%) tested screen-positive (Figure 1; Table 1).

**Table 1:** Comparison of characteristics of participants eligible for sputum collection during 2 national TB prevalence surveys in Vietnam, 2007 and 2017

Characteristic	First survey, 2007				Second survey, 2017			p value†
	Participants	Screened positive*	% Positive		Participants	Screened positive*	% Positive	
Total	94,156	7,529	8.0		61,763	4,595	7.4	<0.001
Sex								
M	42,596	4,580	10.8		27,150	2,794	10.3	0.053
F	51,560	2,949	5.7		34,613	1,801	5.2	0.001
Age group, y								
15–24	20,934	620	3.0		6,542	120	1.8	<0.001
25–34	18,681	950	5.1		10,191	349	3.4	<0.001
35–44	19,790	1,429	7.2		11,508	548	4.8	<0.001
45–54	16,285	1,587	9.8		13,289	1,056	8.0	<0.001
55–64	8,138	1,055	13.0		11,143	1,162	10.4	<0.001
≥65	10,328	1,888	18.3		9,090	1,360	15.0	<0.001
Area								
Urban	26,353	2,058	7.8		18,656	1,383	7.4	0.119
Remote	27,532	2,406	8.7		15,882	1,179	7.4	<0.001
Rural	40,271	3,065	7.6		27,225	2,033	7.5	0.489
Region								
North	45,669	3,913	8.6		25,575	1,849	7.2	<0.001
Central	14,646	1,062	7.3		13,525	1,195	8.8	<0.001
South	33,841	2,554	7.6		22,663	1,551	6.8	0.002

*Screened positive if participants had >1 of self-reported cough for >2 wk, TB treatment <2 y preceding the survey, or abnormal chest radiography images. †By Pearson χ^2^ test to compare characteristics between the first and the second surveys.

Among screen-positive participants in the first survey, 6,780/7,529 (90.1%) had available sputum smear microscopy results; 121 (0.13% of 94,156 total participants) were smear-positive (Table 2). This proportion was similar in the second survey with positive sputum smear results in 77 (0.12% of 61,763 total participants; p = 0.835). There were 218 (0.23%) participants culture-positive for *Mycobacterium tuberculosis* in the first survey and 125 (0.20%) in the second (p = 0.230). The proportion of LJ cultures growing nontuberculous mycobacteria or being contaminated was significantly higher in the second survey than in the first survey (p<0.001; Table 2).

**Table 2:** Results of laboratory testing and TB cases during 2 national TB prevalence surveys in Vietnam, 2007 and 2017*

	First survey, 2007			Second survey, 2017		p value†
	No. participants	% Participants		No. participants	% Participants	
Total participants	94,156	100.0		61,763	100.0	NA
Participants screened positive	7,529	8.0		4,595	7.4	<0.001
DSSM‡						
Negative	6,659	7.07		4,109	6.65	0.001
Any positive	118	0.13		77	0.12	0.835
Positive scanty	35	0.04		36	0.06	0.056
Positive 1+	47	0.05		26	0.04	0.485
Positive 2+	21	0.02		7	0.01	0.114
Positive 3+	15	0.02		8	0.01	0.636
Not reported§	752	0.80		409	0.66	0.003
LJ culture						
MTB growth	218	0.23		125	0.20	0.230
NTM growth	10	0.01		37	0.06	<0.001
No growth	6,513	6.92		3,590	5.81	<0.001
Contaminated	34	0.04		245	0.40	<0.001
Not reported§	754	0.80		598	0.97	<0.001
TB case	218	0.23		125	0.20	0.204
DSSM(+) – LJ(+)	105	0.11		38	0.06	0.001
DSSM(–) – LJ(+)	113	0.12		87	0.14	0.298

*DSSM, direct smear microscopy; LJ, Lowenstein-Jensen; MTB, *Mycobacterium tuberculosis*; NA, not applicable; NTM, nontuberculosis mycobacteria; TB, tuberculosis; +, positive; –, negative.. †Pearson χ^2^ test to compare characteristics between the first and the second surveys. ‡Direct smear microscopy of the morning sample only. §Test result was unknown or no sample available.

Overall, the crude prevalence of culture-positive TB decreased 26.1% (95% CI 7.5%–44.7%) between the 2 surveys (p = 0.007; Appendix Table 5), from 191 (95% CI 167–218) cases/100,000 adults in the first survey to 142 cases/100,000 adults (95% CI 113–169) in the second survey. The crude prevalence of smear-positive TB decreased by 53.0% (95% CI 28.7%–77.2%; Appendix Table 6), from 92 (95% CI 78–125) cases/100,000 adults in 2006–2007 to 43 (95% CI 31–59) cases/100,000 adults in 2017–2018. The crude prevalence of smear-negative TB was 99 (95% CI 82–119) cases/100,000 adults in the first survey and 99 (95% CI 80–122) cases/100,000 adults (p = 0.938) in the second survey (Appendix Table 7).

**Figure 2:**
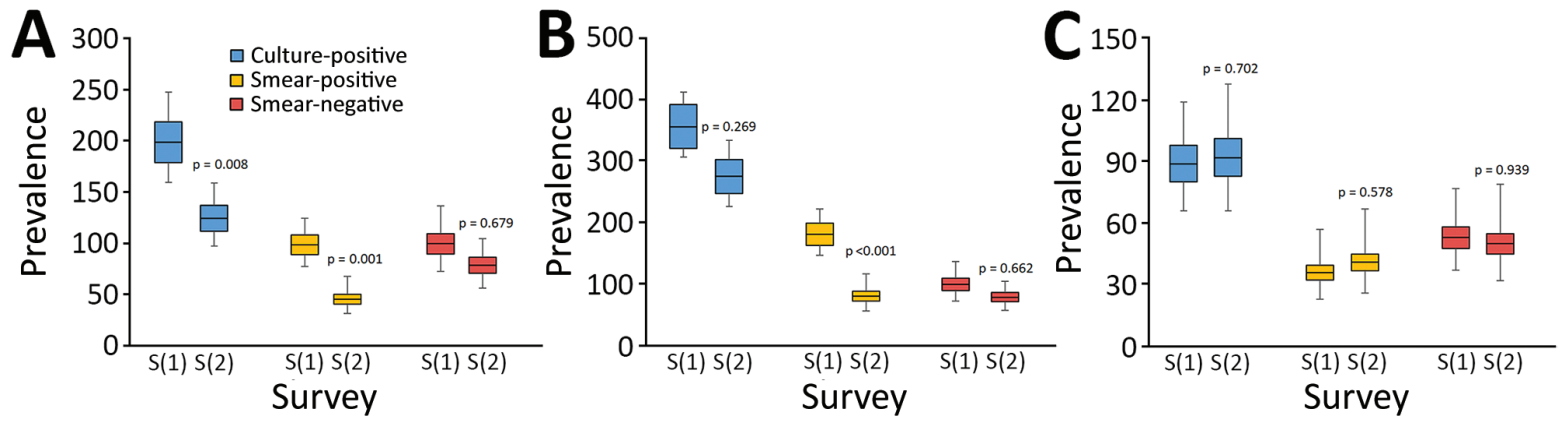
Comparison of the prevalence (cases/100,000 adults) of culture-positive TB, smear-positive TB and smear-negative TB between the first and second national TB prevalence surveys in Vietnam, 2007 and 2017. (A) Overall prevalence; (B) prevalence among male participants; (C) prevalence among female participants. Box tops and bottoms indicate the standard errors of the prevalence; horizontal lines within boxes indicate the point estimates of the prevalence; error bars indicate 95% CIs. S(1), first TB prevalence survey (2007); S(2), second TB prevalence survey (2017).

Sputum smear results, LJ culture results, or both, were missing for 805/7,529 (10.7%) screen-positive participants in the first survey and for 881/4,595 (19.2%) in the second survey. Imputation and adjustment by inverse probability weighting and poststratification resulted in increased adjusted prevalence rates for culture- or smear-positive TB and for smear-negative TB by an average of 11.3% for the first survey and by an average of 20.0% for the second survey. The adjusted prevalence of culture-positive TB declined by 37.1% (95% CI 11.5%–55.4%), from 199 (95% CI 160–248) cases/100,000 adults in 2006–2007 to 125 (95% CI 98–159) cases/100,000 adults in 2017–2018 (p = 0.008; Figure 2; Appendix Table 5). This decline was 53.1% (95% CI 27.0–69.7) for smear-positive, from 99 cases/100,000 adults (95% CI 78–125) to 46 cases/100,000 adults (95% CI 32–68) (p = 0.001; Figure 2; Appendix Table 6). We observed no significant reduction in smear-negative TB prevalence (21.3%, 95% CI: −22.0% to 49.7%; p = 0.679) (Appendix Table 7).

**Figure 3:**
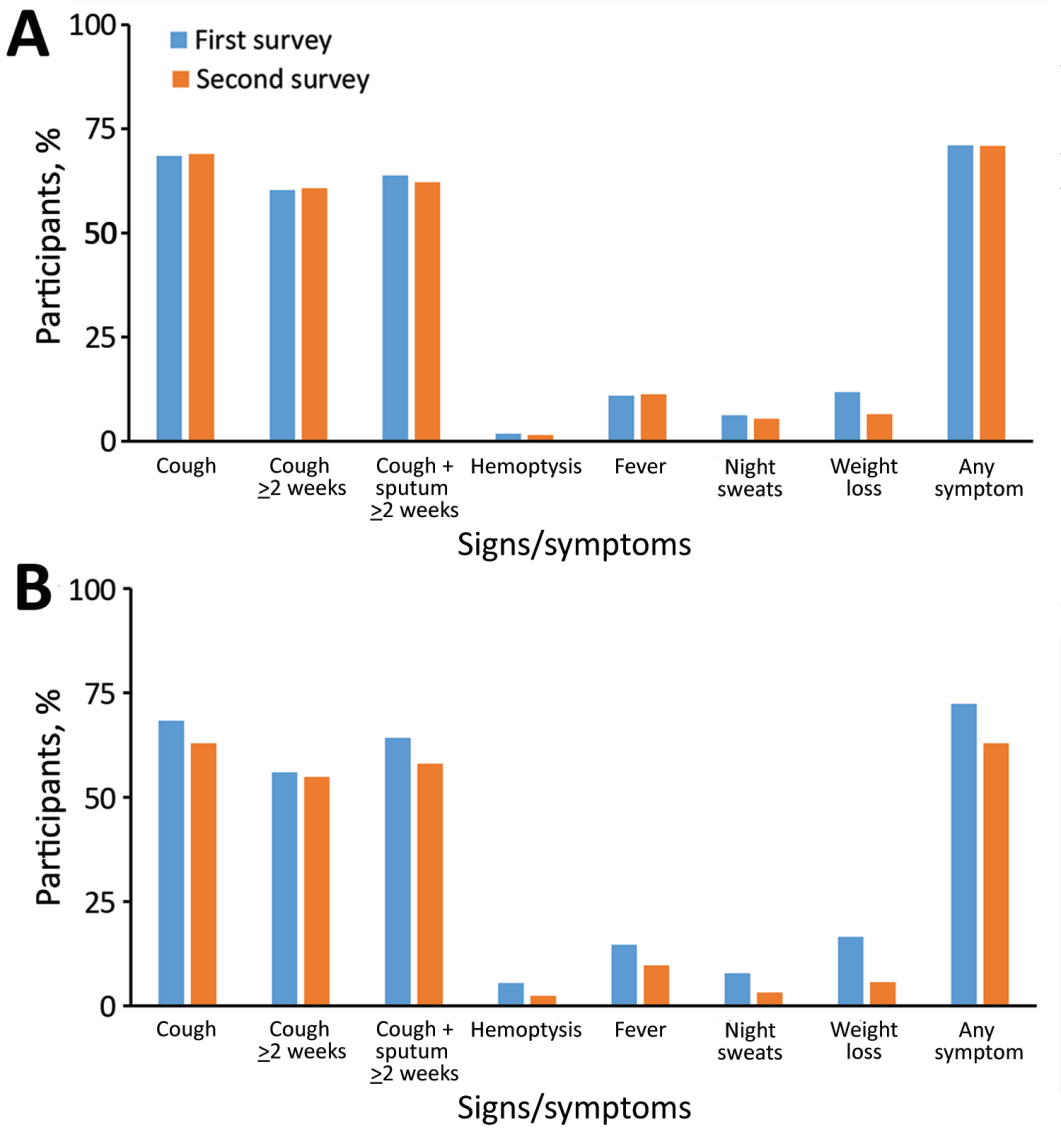
Comparison of symptoms suggestive of TB reported during in-depth interviews of participants who screened positive (A) and survey TB cases (B) identified during the first and second national TB prevalence surveys in Vietnam, 2007 and 2017.

When we stratified results by age and sex, we found a significant reduction (64.0%, 95% CI 40.1%–78.4%; p<0.001) in the prevalence of smear-positive TB among men: 71% (95% CI 32.9%–87.6%; p = 0.026) among men in the 35–44 year age group and 73.8% (95% CI 22.5%–91.1%; p = 0.019) among men ≥65 years of age (Appendix Table 6). The adjusted prevalence of smear-positive TB significantly decreased from the first survey to the second in rural areas (73.5%, 95% CI 35.1%–89.2%; p = 0.017), and in the southern (53.5%, 95% CI 8.3%–76.5%; p = 0.047) and northern (65.2%, 95% CI 20.6%–84.7%; p = 0.022) regions of Vietnam (Appendix Table 6); a significant decline in culture-positive TB (33.3%, 95% CI 7.4%–65.0%; p = 0.023) occurred only in rural areas (Appendix Table 5). In-depth interviews in the 2 surveys showed no difference between the symptoms reported by the screen-positive participants and TB cases found in the 2 surveys, except for self-reported weight loss, which was lower in the second survey (Figure 3; Appendix Table 4).

## Discussion

Our analyses show a 37% decline in the adjusted prevalence of culture-positive TB in Vietnam over a 10-year period between 2006–2007 and 2017–2018, equating to an average annual decline of 4.5%. This decline was mainly due to the 53% reduction in smear-positive TB, particularly because of substantial reductions among men, persons living in rural areas, and persons in the northern and southern parts of the country. We found no reduction in the adjusted prevalence of smear-negative TB. These results are in line with a 57% decline in smear-positive TB prevalence observed from 2000 to 2010 in China (*18*) and declines in culture-positive TB of 45% and smear-positive TB of 38% in Cambodia from 2002 to 2011 (*19*).

The second survey’s participation rate was much lower than that for the first survey (Figure 1), which could be explained by the difference between the household enumeration inclusion criteria of the 2 surveys; in the first survey, only those who confirmed availability to participate in the survey during fieldwork days were recorded as eligible. Also, recent economic growth in Vietnam has enabled more people to access out-of-pocket healthcare services, making attending a TB prevalence survey to receive free health checkups less attractive. The types of symptoms and proportion of participants with any symptoms suggestive of TB among screen-positive participants and culture-positive TB cases in the 2 surveys were similar, except that weight loss was more frequently reported in the first survey. This difference could be attributable to differences in training interviewers; for the first survey the interviewers were explicitly trained to ask carefully about weight loss to help ascertain the prevalence of chronic obstructive pulmonary disease, a secondary objective in the first survey only (H.B. Nguyen, unpub. data). 

Over the decade between surveys, smear-positive TB prevalence declined, but smear-negative TB prevalence remained static. Vietnam, as a low-middle income country, has used sputum smear microscopy as a key method to diagnose TB nationwide (*20*). Microscopy cannot detect smear-negative TB, which might explain why the recorded prevalence of smear-negative TB did not change after the first survey. Of note, whereas the prevalence of smear-positive TB decreased by 64% among men, it did not change among women. This difference may be because for decades women in Vietnam have sought healthcare more often than men and experienced shorter delays (*21*). TB case finding among women, with the support of the Women’s Union, has been instrumental in reducing TB prevalence (*22*). Thus, it may be that, compared with men, women already had relatively shorter TB duration, and so prevalence was less affected by TB control interventions during the decade. In addition, the Global Adult Tobacco Survey conducted in Vietnam in 2015 found that smoking, a well-known risk factor for TB, was more frequent among men (47.4%) than among women (1.4%), although the smoking prevalence among men had declined slightly compared with a similar survey in 2010 (*23*).

The difference in TB prevalence between men and women was also reflected in the decline of the male-to-female ratio in the prevalence of culture-positive TB found in the 2 surveys by using similar methods, from 4.0:1 in 2006–2007 to 3.0:1 in 2017–2018. This ratio is still high compared with the average found in 56 previous TB prevalence surveys in low-middle income countries (2.2:1) (*24*). The consistently high male-to-female ratios in both studies suggest that the difference by sex in recorded TB prevalence reflects an actual difference in disease occurrence, warranting further research.

We cannot be conclusive about the causes of the reduction in TB prevalence in our study because confounding factors may have affected the trend in TB burden in Vietnam. One factor is economic growth in Vietnam during 2007–2017; steady annual GDP growth rates ranged from 5.2% to 7.1% (*25*). When the economy grows, TB burden tends to decline because nutritional, housing, and working conditions improve (*26*). A decline solely due to economic growth would likely have affected smear-positive and smear-negative TB equally. However, many interventions employed by NTP in the 10 years between surveys relied on sputum smear microscopy, which cannot detect smear-negative TB, possibly explaining these divergent trends. The decline in smear-positive TB suggests that the interventions were effective and are at least partially responsible for the observed decline. Nevertheless, estimates of the burden of TB in Vietnam remained high when newer, more advanced, recommended diagnostics were applied to measure prevalence. Data based only on sputum smear microscopy and LJ culture underestimated prevalence by 60%–62% compared with prevalence estimated by using Xpert MTB/RIF rapid molecular assays (Cepheid) and the more sensitive MGIT BACTEC960 liquid culture method (BD) (*12*). It is reasonable to believe that the first survey underestimated the TB prevalence to a similar extent, which may be true for many prevalence surveys using less sensitive methods. This likelihood also underscores the need to replace microscopy with Xpert MTB/RIF as the primary TB diagnostic method for TB care nationwide.

Our study’s first limitation was that we could not address all of the analytic differences in screening and diagnostic technologies used in the 2 surveys. This difference may have affected the comparability of screening and diagnostic results, contributing to uncertainties surrounding the true TB burden in both surveys. For screening, digital chest radiograph was applied in all clusters in the second survey, whereas participants in only two thirds of the clusters in the first survey had digital chest radiograph, which probably resulted in lower sensitivity of TB screening in the first survey (Appendix Table 2). For diagnosis, sputum processing using harsher decontamination methods during the first survey might have resulted in more false-negative culture results compared with the second survey. The difference in sputum processing could also explain the higher rate of contamination in the second survey. Second, the comparison of TB prevalence between surveys among participants in the youngest age group (15–24 years of age) was not available because no culture-positive TB case was found in the second survey among this age group. This lack of results might be due to undersampling in this age group because the time they needed to go to school or work interfered with the timing of fieldwork, as described elsewhere (*12*). We attempted to correct for this lack of data using multiple imputation and poststratification as recommended (*13*).

In summary, our study shows a statistically significant reduction in prevalence of smear-positive TB in Vietnam between 2007 and 2017 but no statistically significant reduction in prevalence of smear-negative TB. These results should be used by the NTP to direct efforts and resources to support TB prevention and control nationwide. In particular, our results suggest that replacing microscopy with rapid molecular diagnostic methods, such as Xpert MTB/RIF, and screening for TB using digital chest radiograph to enhance rapid case finding and treatment could further reduce the TB burden in Vietnam. 

Appendix Additional methods and results comparisons between the first and second National TB Prevalence surveys in Vietnam
