# Supplementary material for: Decline of Tuberculosis Burden in Vietnam Measured by Consecutive National Surveys, 2007–2017
Source: Emerg Infect Dis. 2021 Mar;27(3):872–9. doi: 10.3201/eid2703.204253 (PMC7920672; doi:10.3201/eid2703.204253)
Supplement: Appendix — Additional methods and results comparisons between the first and second National TB Prevalence surveys in Vietnam [file 20-4253-Techapp-s1.pdf]

Article DOI: https://doi.org/10.3201/eid2703.204253

Decline of Tuberculosis Burden in Vietnam Measured by Consecutive National Surveys, 2007–2017

Appendix

Appendix Table 1. Comparison of sampling characteristics between the first and second National TB Prevalence surveys in Vietnam

Characteristics	First survey (2007)	Second survey (2017)
Sampling frame	Multistage sampling: Districts selected proportional to population size, then communes selected proportional to population size, then subcommunes selected by simple random sampling, and including all households in the selected subcommune. *Stratification by area (urban/rural/remote)**	Multistage sampling: Districts selected proportional to population size, then communes selected proportional to population size, then subcommunes selected by simple random sampling, and including all households in the selected subcommune. *No stratification**
Enumeration	All who have lived in the household for *≥3 months** Military camps and prisons excluded	All who have lived in the household for *≥2 weeks** Military camps and prisons excluded
Eligibility	All who are mentally able to understand the study procedures and the risks and benefits of participation, *and confirmed that they were available to attend the survey field procedures**	All who are mentally able to understand the study procedures and the risks and benefits of participation

*Italics indicate differences in sampling characteristics between first and second surveys

Appendix Table 2. Comparison of screening and diagnosis procedures between the first and second national TB prevalence surveys in Vietnam

Methodology	First survey (2007)	Second survey (2017)
Screening interview	Questions on cough and TB treatment history	Questions on cough and TB treatment history
Chest radiography	Digital for 42/70 clusters, *70x70mm photofluorography film for the other clusters**	Digital for all cluster
Sputum smear microscopy	*2 spot samples in local district laboratories by field laboratory teams** 1 morning sample in central laboratories	*No spot samples** 1 morning sample in central laboratories
Culture	*No MGIT applied** LJ on morning sample after smear (1 slope), using modified Petrov method with *4% NaOH** for decontamination	*MGIT on morning sample after smear in all clusters (1 tube) and** LJ on the same sample (after MGIT) in 72/82 clusters (1 slope), using *3% NaOH** for decontamination.

*Italics indicate differences in methodology between first and second surveys

Appendix Table 3. Criteria of participants eligible for sputum examination during the first and second national TB prevalence surveys in Vietnam

Criterion	First survey (2007)	Second survey (2017)	Definition used for comparison between surveys
Symptom screening	Cough ≥2 weeks *with sputum**	Cough ≥2 weeks	Cough ≥2 weeks with sputum
Pregnancy	*Not available**	Cough <2 weeks and pregnant	Not included as eligible for sputum examination
History of TB treatment	Current TB treatment or TB treatment ≤2 y preceding the survey	Current TB treatment or TB treatment ≤2 y preceding the survey	No adaptation needed
Chest x-ray screening	Chest x-ray suggesting TB	Chest x-ray suggesting TB	No adaptation needed

*Italics indicate differences in eligibility criteria between first and second surveys

Appendix Table 4. Comparison of symptoms consistent with TB reported during the in-depth interview by participants who initially screened positive for TB and survey TB cases found during the first and second national TB prevalence surveys in Vietnam

	First survey (2007)		Second survey (2017)		
Characteristic	n	%	n	%	p-value*
Screened positive participants	7,529		4,595		
Cough	5,158	68.5	3,170	69.0	0.581
Cough ≥2 weeks	4,541	60.3	2,791	60.7	0.641
Cough with sputum	4,803	63.8	2,856	62.2	0.070
Hemoptysis	135	1.2	68	1.5	0.192
Fever	821	10.9	517	11.3	0.554
Weight loss	887	11.8	299	6.5	<0.001
Night sweats	470	6.2	249	5.4	0.063
Any symptoms	5,352	71.1	3,258	71.9	0.830
Culture-positive TB cases	218		125		
Cough	149	68.4	79	63.2	0.331
Cough ≥2 weeks	122	56.0	69	55.2	0.891
Cough with sputum	140	64.2	73	58.4	0.285
Hemoptysis	12	5.5	3	2.4	0.176
Fever	32	14.7	12	9.6	0.176
Weight loss	36	16.5	7	5.6	0.003
Night sweats	17	7.8	4	3.2	0.087
Any symptoms	157	72.0	79	63.2	0.090

*p-value ≤0.05 is statistically significant

Appendix Table 5. Comparison of prevalence per 100,000 adults of culture-positive tuberculosis in the first and second national TB prevalence surveys in Vietnam*

	First survey (2007)			Second survey (2017)			Comparison					
							Crude % change‡			Adjusted % change§		
Characteristic	No.	Crude point estimate (95%CI)	Adjusted† point estimate (95%CI)	No.	Crude point estimate (95%CI)	Adjusted† point estimate (95%CI)	%	95%CI, %	p-value¶	%	95%CI, %	p-value¶
Overall	218	191 (167–218)	199 (160–248)	125	142 (113–169)	125 (98–159)	–26.1	−44.7 to −7.5	0.007	–37.1	−55.4 to −11.5	0.008
Sex												
M	171	320 (276–372)	356 (307–412)	92	221 (180–271)	275 (226–334)	–31.1	−51.6 to −10.5	0.004	–18.0	−42.2 to +16.3	0.269
F	47	77 (58–103)	89 (66–119)	33	71 (51–100)	92 (66–128)	–10.4	−52.6 to +31.7	0.631	+11.1	−96.1 to +36.7	0.702
Age group, y												
15–24	9	32 (17–62)	40 (21–76)	0	0	0	NA	NA	NA	NA		NA
25–34	22	91 (60–138)	102 (68–154)	12	68 (39–120)	77 (44–135)	–25.0	−84.5 to +34.5	0.422	+19.0	−50.2 to +194.1	0.687
35–44	48	207 (156–275)	224 (169–297)	21	122 (80–188)	143 (94–217)	–40.9	−78.8 to −3.0	0.044	–20.6	−49.3 to +57.8	0.473
45–54	50	271 (205–357)	304 (233–397)	29	169 (118–243)	209 (149–294)	–37.5	−73.3 to −1.7	0.044	–20.6	−33.3 to +54.8	0.384
55–64	28	314 (217–454)	353 (245–509)	35	265 (191–373)	344 (252–469)	–15.3	−61.7 to +31.1	0.512	+7.5	−72.4 to +32.9	0.772
≥65	61	534 (416–686)	597 (469–760)	28	258 (178–370)	346 (244–490)	–53.5	−84.0 to −22.9	0.001	–34.6	−8.7 to +60.6	0.098
Area												
Urban	59	181 (140–234)	202 (157–261)	55	193 (148–252)	232 (181–297)	+4.9	−32.9 to +42.7	0.800	+31.6	−117.4 to +20.6	0.273
Remote	52	164 (125–216)	178 (136–234)	23	111 (74–167)	160 (111–229)	–32.4	−71.1 to +6.3	0.118	–1.0	−61.3 to +50.2	0.995
Rural	107	214 (177–259)	243 (201–294)	47	121 (91–162)	150 (113–199)	–43.4	−68.3 to −18.5	0.001	–43.2	−65.0 to −7.4	0.023
Zone												
North	102	189 (155–229)	212 (175–257)	38	117 (85–161)	146 (108–197)	–39.5	−67.0 to −12.1	0.009	–33.3	−8.7 to +58.8	0.101
Central	23	130 (86–195)	166 (110–252)	24	138 (92–205)	174 (118–256)	+6.2	−52.7 to +65.1	0.837	–4.8	−100.0 to +54.3	0.894
South	93	219 (179–269)	235 (192–288)	63	166 (130–212)	209 (165–266)	–24.5	−52.0 to +3.1	0.085	+2.0	−69.5 to +38.7	0.939

*NA, not available.
†Weighted prevalence adjusted based on multiple imputation of missing values, inverse probability weighting and post-stratification.
‡Proportion change of the crude prevalence in the second survey compared with the first survey.
§Proportion change of the weighted prevalence in the second survey compared with the first survey; **+,** increase; **–,** decrease.
¶P-value of corrected Pearson's chi-squared test to compare characteristics between the first survey and the second survey.

Appendix Table 6. Comparison of prevalence per 100,000 adults of smear-positive tuberculosis in the first survey and the second national TB prevalence surveys in Vietnam*

	First survey (2007)			Second survey (2017)			Comparison					
							Crude % change‡			Adjusted % change§		
Characteristic	No.	Crude point estimate (95%CI)	Adjusted† point estimate (95%CI)	No.	Crude point estimate (95%CI)	Adjusted† point estimate (95%CI)	%	95%CI, %	p-value¶	%	95%CI, %	p-value¶
Overall	105	92 (76–111)	99 (78–125)	38	43 (31–59)	46 (32–68)	–53.0	−77.2 to −28.7	<0.001	–53.1	−69.7 to −27.0	0.001
Sex												
Male	86	161 (130–199)	181 (147–222)	24	58 (39–86)	81 (56–117)	–64.2	−89.7 to −38.7	<0.001	–64.0	−78.4 to −40.1	<0.001
Female	19	31 (20–49)	36 (23–57)	14	30 (18–51)	41 (26–67)	–3.1	−70.9 to +64.7	0.929	+4.2	−53.9 to +132.6	0.578
Age group												
15–24	5	18 (7–43)	22 (9–52)	0	0	0	NA	NA	NA	NA		NA
25–34	9	37 (19–71)	42 (22–79)	8	45 (23–91)	48 (24–95)	22.2	−84.7 to +129.1	0.680	+13.6	−58.5 to +212.5	0.248
35–44	29	125 (87–180)	133 (93–191)	6	35 (16–78)	42 (20–90)	–72.0	−114.7 to −29.4	0.004	–71.2	−87.6 to −32.9	0.026
45–54	27	146 (100–213)	163 (112–237)	11	64 (36–116)	88 (52–149)	–56.1	−101.9 to −10.4	0.021	–51.5	−76.8 to +2.0	0.235
55–64	11	123 (68–222)	147 (84–260)	10	76 (41–141)	115 (67–199)	–38.4	−108.7 to +31.9	0.267	–24.8	−68.9 to +81.8	0.737
≥65	24	210 (141–313)	244 (167–357)	3	27 (9–85)	62 (26–149)	–86.9	−129.5 to −44.2	0.001	–73.8	−91.1 to −22.5	0.019
Area												
Urban	33	101 (72–142)	112 (80–158)	23	81 (54–122)	99 (67–147)	–20.1	−67.3 to +27.1	0.408	–35.5	−67.3 to +26.6	0.870
Remote	22	70 (46–106)	77 (50–116)	6	29 (13–65)	56 (30–103)	–58.3	−111.8 to −4.9	0.057	–46.2	−72.9 to +7.5	0.587
Rural	50	100 (75–132)	115 (88–151)	9	23 (12–45)	34 (19–61)	–76.8	−108.4 to −45.2	<0.001	–73.5	−89.2 to −35.1	0.017

	First survey (2007)			Second survey (2017)			Comparison					
							Crude % change‡			Adjusted % change§		
Characteristic	No.	Crude point estimate (95%CI)	Adjusted† point estimate (95%CI)	No.	Crude point estimate (95%CI)	Adjusted† point estimate (95%CI)	%	95%CI, %	p-value¶	%	95%CI, %	p-value¶
Zone												
North	46	85 (64–114)	97 (73–130)	5	15 (6–37)	31 (15–62)	–81.9	−114.8 to −48.9	<0.001	–65.2	−84.7 to −20.6	0.022
Center	16	90 (55–147)	111 (68–180)	11	63 (35–114)	80 (46–139)	–30.0	−94.1 to +34.1	0.362	–39.4	−77.2 to +61.3	0.444
South	43	101 (75–137)	108 (80–146)	22	58 (38–88)	76 (51–111)	–43.0	−81.2 to −4.7	0.032	–53.5	−76.5 to −8.3	0.047

*NA, not available.
†Weighted prevalence adjusted based on multiple imputation of missing values, inverse probability weighting and post-stratification.
‡Proportion change of the crude prevalence in the second survey compared with the first survey.
§Proportion change of the weighted prevalence in the second survey compared with the first survey; **+,** increase; **–,** decrease.
¶P-value of corrected Pearson's chi-squared test to compare characteristics between the first survey and the second survey.

Appendix Table 7. Comparison of prevalence per 100,000 adults of smear-negative tuberculosis in the first survey and the second national TB prevalence surveys in Vietnam.

	First survey (2007)			Second survey (2017)			Comparison					
							Crude % change‡			Adjusted % change§		
Characteristic	No.	Crude point estimate (95%CI)	Adjusted† point estimate (95%CI)	No.	Crude point estimate (95%CI)	Adjusted† point estimate (95%CI)	%	95%CI, %	p-value¶	%	95%CI, %	p-value¶
Overall	113	99 (82–119)	100 (73–137)	87	99 (80–122)	79 (59–105)	–1.1	–29.0 to 26.7	0.938	–21.3	−49.7 to 22.0	0.679
Sex												
Male	85	159 (129–197)	175 (141–216)	68	163 (129–207)	194 (153–246)	+ 2.5	–29.8 to 34.8	0.878	–24.8	−51.9 to 19.0	0.662
Female	28	46 (32–67)	53 (37–77)	19	41 (26–64)	50 (32–79)	–15.4	–69.3 to 38.4	0.579	–23.7	−65.6 to 69.5	0.939
Age group												
15–24	4	14 (5–38)	17 (6–47)	0	0	0	NA	NA	NA	NA	NA	NA
25–34	13	54 (31–92)	61 (36–102)	4	23 (9–60)	29 (11–76)	–57.7	–126.0 to 20.6	0.579	–55.8	−91.2 to 122.2	0.648
35–44	19	82 (52–128)	90 (57–142)	15	87 (23–145)	100 (60–166)	+ 6.6	–63.6 to 76.9	0.132	–8.3	−61.7 to 122.2	0.428
45–54	23	124 (83–187)	141 (93–212)	18	105 (66–167)	121 (76–191)	–15.7	–72.1 to 40.7	0.852	–21.9	−65.2 to 72.4	0.915
55–64	17	190 (118–306)	206 (128–331)	25	190 (128–280)	228 (155–336)	–0.3	–61.8 to 61.1	0.588	+ 29.9	−32.0 to 150.0	0.528
≥65	37	324 (235–447)	353 (258–483)	25	230 (156–340)	282 (192–414)	–31.8	–74.0 to 10.3	0.991	–16.0	−56.3 to 61.3	0.728
Area												
Urban	26	80 (54–117)	90 (61–131)	32	112 (79–159)	132 (94–185)	+ 36.6	–24.9 to 98.2	0.24	+ 3.1	−52.8 to 127.3	0.188
Remote	30	95 (66–136)	102 (71–145)	17	82 (71–132)	103 (64–166)	–13.4	–67.9 to 41.1	0.636	–25.4	−70.3 to 104.1	0.842
Rural	57	114 (88–148)	128 (98–166)	38	98 (51–135)	116 (84–160)	–14.0	–51.7 to 23.7	0.47	–38.3	−63.9 to 6.4	0.327
Zone												
North	56	104 (80–135)	114 (88–149)	33	102 (72–143)	114 (82–160)	–4.8	–46.9 to 37.4	0.826	–23.1	−62.1 to 56.3	0.734
Center	7	39 (19–82)	55 (27–113)	13	75 (43–128)	93 (54–160)	+ 89.0	–37.6 to 215.6	0.174	+ 35.1	−50.7 to 270.4	0.320
South	50	118 (89–156)	127 (96–167)	41	108 (79–146)	133 (97–182)	–8.6	–47.9 to 30.8	0.671	–28.1	−62.3 to 37.0	0.582

*NA, not available.
†Weighted prevalence adjusted based on multiple imputation of missing values, inverse probability weighting and post-stratification.
‡Proportion change of the crude prevalence in the second survey compared with the first survey.
§Proportion change of the weighted prevalence in the second survey compared with the first survey; **+,** increase; **–,** decrease.
¶P-value of corrected Pearson's chi-squared test to compare characteristics between the first survey and the second survey.
